# Outcome of patients in acute poisoning with ethylene glycol - factors which may have influence on evolution

**Published:** 2014

**Authors:** A Tanasescu, RA Macovei, MS Tudosie

**Affiliations:** * ”Carol Davila” University of Medicine and Pharmacy, Bucharest, Romania; ** Emergency Hospital Bucharest, Romania; *** Army Center of Medical Research Bucharest, Romania

**Keywords:** acute poisoning with ethylene glycol, glycolic acid, oxalic acid, ethanol, hemodialysis

## Abstract

**Introduction.** Intoxication with ethylene glycol occurs as a result of intentional ingestion in suicide attempts or accidentally. Clinical ethylene glycol poisoning is not specific and occurs in many poisoning cases therefore the diagnosis is difficult. Early diagnostic and establishment of therapy are very important for a favorable evolution. The mortality rate of ethylene glycol intoxication ranges between 1 and 22% depending on the amount of alcohol ingestion and the time period between alcohol ingestion and initiation of therapy.

**Methods.** Retrospectively analyzed data from 18 patients admitted with ethylene glycol poisoning in the emergency department between 2011 and 2012. The following were taken into consideration: incidence of intoxication in the group study, medical history, the amount ingested and the time since the ingestion of ethylene glycol and the admission to hospital, presence of metabolic acidosis and laboratory test results on admission (urea, creatinine osmolar or anion gaps), the treatment initiated and the outcome of the patient.

**Results.** 18 patients with ethylene glycol intoxication were admitted to hospital between 2011 and 2012. The initial diagnosis based on a detailed clinical history in combination with the presence of metabolic acidosis with elevation of the osmolar or anion gaps. 12 of the 18 patients were man (66%) and age range interval was between 23 and 77 years. The time from the ingestion of ethylene glycol and the admission to hospital was between 30 minutes and older than 24 hours. 14 patients have been presented earlier to the hospital, between 30 minutes and 12 hours (in the first part of the clinical stage) and 13 of the 14 patients had a favorable evolution. One of these patients had an unfavorable evolution. Regarding this patient, the amount ingested was unknown. 10 of the 18 patients had a voluntary ingestion (55,55%) and 6 of the 18 patients had an alcoholism medical history. The amount ingested by the patients was between 20 ml and 500 ml. Metabolic acidosis was present up to 55,55% (10 of the 18 patients) in the blood gas analysis on admission, with pH on admission between 6.9 and 7.27, with anion gap ranging between 16.3 mmol/l and 32.6 mmol/l (normal range 8-16 mmol/l). Ten patients also had an increased level of urea and creatinine with a level between 1.24 to 6.85 mg/dl for creatinine (normal range 0.5-1.2 mg/dl) and 49 to 98 mg/dl for urea (normal range 15-43 mg/dl) and developed acute kidney injury that required regular HD sessions. Mechanical ventilation was required for 7 of the 18 patients (38.88%). Five patients died (27.77%). Although metabolic acidosis was corrected under hemodialysis, there were patients who had multiple organ failure and systems: acute respiratory failure requiring ventilator support, acute renal failure requiring dialysis daily sessions, altered state of consciousness.

**Conclusions.** The early diagnostic and exclusion of the other diseases and other poisoning led to a specific treatment of the intoxication. The time from the ingestion of ethylene glycol and the early establishment of therapy is very important for a favorable evolution and can prevent substantial mortality.

## Introduction

Intoxication with ethylene glycol occurs as a result of intentional ingestion in suicide attempts or accidentally. The mortality rate of ethylene glycol intoxication ranges between 1 and 22% depending on the amount of alcohol ingestion and the time period between the alcohol ingestion and the initiation of therapy [**1**,**2**]. The early diagnostic and establishment of therapy are very important for the evolution of patients on ethylene glycol poisoning. 

Ethylene glycol is used as antifreeze and as a solvent for a variety of products. In biological fluids, ethylene glycol can be easily determined by gas chromatography. Many hospital laboratories do not have the ability to perform this blood test and, in the absence of this test, the diagnosis must be made based on the clinical examination and patient history collaborated with laboratory test results on admission: pH, anion gap, cHCO3.

The accumulation of toxic metabolites, such as glycolic acid and oxalic acid is responsible for toxicity on intoxication with ethylene glycol. The metabolic acidosis is a result of accumulation of glycolic acid and lactic acid. In addition, the inhibition of Krebs cycle and the accumulation of oxalic acid have a contribution to the metabolic acidosis [**3**].

After ingestion, ethylene glycol is rapidly absorbed and distributed in all tissues of the body with a volume of distribution of 0.7-0.8 l / k [**4**,**5**].

The first step in the metabolism of ethylene glycol is its conversion to glycolaldehyde by the action of alcohol dehydrogenase. Glycolaldehyde is metabolized to glycolic acid by aldehyde dehydrogenase. Both oxidative processes lead to the formation of NADH from NAD, thereby changing the redox potential and promoting the production of lactate and pyruvate. Glycolic acid is further metabolized to glyoxylic acid and then to oxalic acid [**3**,**6**]. The accumulation of glycolic acid in the body is mainly responsible for toxicity [**7**].

Chelation of oxalic acid with calcium ions forms insoluble calcium oxalate, which may lead to hypocalcaemia and nephrotoxicity and neurotoxicity [**8**].

Plasma half-lives of ethylene glycol are of approximately 3-5 hours. At levels of Ethanol of 100-200 mg / dl, plasma half-lives of ethylene glycol are extended to 17 hours due to an increased affinity of ethanol for alcohol dehydrogenase. The clinical signs of intoxication begin after a period of about 4 hours from ingestion. Often, these signs and symptoms are classified into three stages for a didactic purpose. Ethylene glycol poisoning does not often have the clinical picture of all stages, some are more pronounced than others. **Stage I** (first 1-12 hours post ingestion): initial depression of the central nervous system is very similar to ethanol intoxication. The patient experiences dizziness, psychomotor agitation, nystagmus, tachycardia, hypertension and vomiting. In severe cases coma and convulsions occur. Hyperventilation increases as the results of metabolic acidosis intensify [**9**]. **Stage II** (the first 12-24 hours post ingestion) cardiopulmonary phase develops in approximately 24 hours after the ingestion and is characterized by cardiopulmonary failures. Dyspnea, hyperventilation, tachycardia, cyanosis, hypertension are clinical signs typical of this stage. Because oliguria is present, the patient may also present pulmonary edema at this stage. Chest X-ray shows massive bilateral infiltration at this stage [**9**]. If the status of the patient is critical, the impairment can be fatal [**10**]. **Stage III** (after 24-72 hours from onset): oliguria that develops gradually appears, especially if the treatment is not applied correctly and on time. The urinary sediment contains at most patients with calcium oxalate crystals, which, on optical microscope, have the appearance of needles. Acute renal failure may be reversible under the proper treatment, but most patients require temporary dialysis for 2 to 3 weeks [**10**]. Prognosis of renal failure without other underlying conditions is good, although some patients require hemodialysis for a longer period [**9**,**11**].

## Material and Methods

Data from 18 patients with ethylene glycol poisoning admitted in the emergency department between 2011 and 2012 were retrospectively analyzed. The following were taken into consideration: incidence of intoxication in the group study, medical history, the amount ingested and the time since the ingestion of ethylene glycol and the admission to hospital, presence of metabolic acidosis and laboratory test results on admission – **Table 2** (urea, creatinine osmolar or anion gaps), the treatment initiated and the outcome of the patients - **Table 1**.

## Results

18 patients with ethylene glycol intoxication were admitted to hospital between 2011 and 2012. The initial diagnosis was based on a detailed clinical history in combination with the presence of metabolic acidosis with the elevation of the osmolar or anion gaps. Out of 18 patients, 12 were man (66%) and the age range interval was between 23 and 77 years. The time from the ingestion of ethylene glycol and the admission to hospital was between 30 minutes and older than 24 hours. 14 patients presented earlier to the hospital, between 30 minutes and 12 hours (in the first period of the clinical stage) and 13 out of 14 patients had a favorable evolution. One of these patients had an unfavorable evolution. The amount ingested by this patient was unknown. 16,66% of the patients (3 patients) presented later to the hospital, more than 24 hours after the ingestion of ethylene glycol. 10 out of 18 patients presented a voluntary ingestion (55,55%) and 6 out of 18 patients presented an alcoholism medical history. The amount ingested by patients was between 20 ml and 500 ml. The metabolic acidosis was present in up to 55,55% (10 out of 18 patients) in the blood gas analysis on admission, with a pH between 6.9 and 7.27 on admission and anion gap ranges between 16.3 mmol/l and 32.6 mmol/l (normal range 8-16 mmol/l). Ten patients also had an increased level of urea and creatinine, with a level between 1.24 to 6.85 mg/dl for creatinine (normal range 0.5-1.2 mg/dl) and 49 to 98 mg/dl for urea (normal range15-43 mg/dl) and developed acute kidney injury, which required regular HD sessions. The mechanical ventilation was required for 7 out of 18 patients (38.88%). Five patients died (27.77%). Although the metabolic acidosis was corrected under hemodialysis, patients experienced multiple organ failure and systems: acute respiratory failure requiring ventilator support, acute renal failure requiring dialysis sessions daily, altered state of consciousness.

**Table 1:** Clinical data of patients intoxicated with ethylene glycol

Case	Sex/Age	Voluntary/ Accidentally intoxication	Time since ingestion	Quantity ingested	Treatment	Outcome
1	M/60	Voluntary	6 hours	unspecified	Hemodialysis Ethanol Ventilation support	Death
2	M/53	Accidental	3 hours	100 ml	Ethanol	Recovered
3	M/23	Accidental	1 hour	20-30 ml	Ethanol	Recovered
4	M/43	Accidental	3 hours	50 ml	Ethanol	Recovered
5	F/77	Accidental	7 hours	200 ml	Hemodialysis Ethanol	Recovered
6	M73	Accidental	10 hours	200 ml	Ethanol	Recovered
7	M/52	Voluntary	8 hours	Unknown	Hemodialysis Ethanol Ventilation support	Recovered
8	M/47	Voluntary	4 hours	100 ml	Hemodialysis Ethanol	Recovered
9	F/52	Voluntary	30 minutes	20 ml	Ethanol	Recovered
10	F/77	Voluntary	Unknown	Unknown	Hemodialysis Ethanol Ventilation support	Death
11	F/56	Accidental	1 hour	50 ml	Ethanol	Recovered
12	M/44	Unknown	> 24 hours	150 ml	Hemodialysis Ethanol Ventilation support	Death
13	F/25	Accidental	4 hours	100 ml	Hemodialysis Ethanol	Recovered
14	M/37	Voluntary	4 hours	100 ml	Hemodialysis Ethanol Ventilation support	Recovered
15	M/23	Voluntary	> 24 hours	Unknown	Hemodialysis Ethanol Ventilation support	Death
16	M/32	Voluntary	> 12 hours	30 ml	Hemodialysis Ethanol	Recovered
17	M/50	Voluntary	8 hours		Hemodialysis Ethanol Ventilation support	Death
18	F/31	Voluntary	2 hours		Ethanol	Recovered

**Table 2:** Laboratory test results on admission in intoxicated patients

Case	Sex/Age	Time since ingestion	Quantity ingested	pH	cHCO3	Anion Gap	Creatinine	Urea	Outcome
1	M/60	6 hours	unspecified	6.75	4.6	26.8	1.24	27.0	Death
2	M/53	3 hours	100 ml	7.36	24.3	4.1	0.96	23.7	Recovered
3	M/23	1 hour	20-30 ml	7.41	25.9	3.9	0.90	43.5	Recovered
4	M/43	3 hours	50 ml	7.41	23.3	6.6	1.2	35	Recovered
5	F/77	7 hours	200 ml	7.19	12.6	26.0	0.43	28	Recovered
6	M73	10 hours	200 ml	7.25	14.0	22.7	1.0	22	Recovered
7	M/52	8 hours	Unknown	7.26	19.2	15.3	1.34	49	Recovered
8	M/47	4 hours	100 ml	7.19	13.4	16.3	1.80	40	Recovered
9	F/52	30 min	20 ml	7.42	19.5	16.8	0.83	16.8	Recovered
10	F/77	Unknown	Unknown	7.27	13.4	23.3	1.70	50	Death
11	F/56	1 hour	50 ml	7.42	25.5	8.5	1.09	40	Recovered
12	M/44	> 24 hours	150 ml	6.9	9.3	28.8	2.20	55	Death
13	F/25	4 hours	100 ml	7.23	10.4	20.9	0.94	33	Recovered
14	M/37	4 hours	100 ml	7.10	9.7	23.4	1.13	29	Recovered
15	M/23	> 24 hours	Unknown	6.93	6.5	32.6	6.03	67.8	Death
16	M/32	> 12 hours	30 ml	7.39	16	9.2	6.85	98	Recovered
17	M/50	8 hours	500 ml	7.41	13.2	11.6	1.20	58	Death
18	F/31	2 hours	30 ml	7.42	24.8	9.8	0.75	31.6	Recovered

## Discussion

The cases presented a clinical evolution of the patients with acute poisoning with ethylene glycol which was influenced by the time elapsed from the ingestion of toxins until the establishment of the specific treatment. The outcome was related to an early diagnostic and exclusion of the other diseases and other poisoning correlated with a specific treatment of intoxication.

The goals of the treatment of acute intoxication with ethylene glycol presuppose the initial stabilization and reducing the occurrence of toxic metabolites, such as glycolic acid and oxalic acid.

In ethylene glycol intoxication, hemodialysis may increase the cleansing of toxic metabolites of ethylene glycol, and establish and restore the ionic balance with a favorable evolution. It has been shown to be highly effective in the removal of ethylene glycol and its metabolites from the blood. It was recognized that hemodialysis is effective if an increase eliminating the toxic substances was applied in at least 30% compared to the usual clearance. The characteristics that make hemodialysis effective are the following: molecular mass of toxins <500, hydro solubility, low volume of distribution (<1 L / kgb.w), low binding to serum albumin. Hemodialysis is used to enhance the removal of unmetabolized ethylene glycol, as well as its metabolites from the body [**12**,**13**]. Hemodialysis also has the added benefit of correcting the other metabolic derangements or supporting the deteriorating kidney function. Hemodialysis is usually indicated in patients with severe metabolic acidosis (blood pH less than 7.3), kidney failure, severe electrolyte imbalance, or if the patient’s condition is deteriorating despite the treatment [**14**].

In our cases, metabolic acidosis was present in up to 55,55%, 10 out of 18 patients presenting an alteration of the pH in the blood gas analysis on admission. The patients also had an increased level of urea and creatinine (38.88%), with a level between 1.24 to 6.85 mg/dl for creatinine (normal range 0.5-1.2 mg/dl) and 49 to 98 mg/dl for urea (normal range 15-43 mg/dl) and developed acute kidney injury, which required regular HD sessions. Although the metabolic acidosis can be corrected under hemodialysis, the outcome of the patient may be unfavorable. Five patients (27.77%) had an unfavorable outcome and died. Patients had multiple organ failure and systems: acute respiratory failure requiring ventilator support, acute renal failure requiring dialysis sessions daily, altered state of consciousness. For these patients, the time from the ingestion of ethylene glycol and the admission to hospital was between 6 hours and older than 24 hours and the quantity ingested started at an amount that could not be specified, of up to 500 ml.

Reports of fatalities following the ingestion of ethylene glycol indicate that a volume of 150–1,500 mL consumed at one time may cause death. In humans, the lethal dose of ethylene glycol is estimated to be in the range of 1,400–1,600 mg/kg. The orally lethal dose in humans has been reported to be of approximately 1.4 mL/kg of pure ethylene glycol [**15**]. Based on these estimates, it appears that humans may be more susceptible to the acute lethality of ingested ethylene glycol than the other species. The oral doses of ≥4,000 mg/kg are needed to cause death in laboratory animals (rats, mice, monkeys). However, difficulties in quantifying the amounts consumed by persons who have succumbed to the toxic effects lead to uncertainty in the human lethal dose estimates [**16**].

Another method to reduce the toxic metabolites is the administration of an antidote; in this case, the most suitable for ethylene glycol is the administration of Ethanol, which is most effective if instituted early. Ethanol acts by competing with ethylene glycol for alcohol dehydrogenase, the first enzyme in the degradation pathway. Because ethanol has a much higher affinity for alcohol dehydrogenase, about a 100-times greater affinity, it successfully blocks the breakdown of ethylene glycol into glycolaldehyde, which prevents the further degradation [**15**]. Without the oxalic acid formation, the nephrotoxic effects can be avoided, but the ethylene glycol is still present in the body. It is eventually excreted in the urine, but supportive therapy for the CNS depression and metabolic acidosis will be required until the ethylene glycol concentrations fall below the toxic limits. Ethanol is readily available in most hospitals, it is inexpensive, and can be administered orally as well as intravenously. Ethanol is usually given intravenously as a 5 or 10% solution in 5% dextrose, but it is also sometimes given orally in the form of a strong spirit such as whisky, vodka, or gin. Patients receiving ethanol therapy also require frequent blood ethanol concentration measurements and dosage adjustments to maintain a therapeutic ethanol concentration. The patient in our retrospective study received ethanol during the treatment as method of reducing of the nephrotoxic effect of metabolites of ethylene glycol. 6 out of 18 patients had an alcoholism medical history and they concomitantly consumed ethanol with ethylene glycol. For these patients, the nephrotoxic effects by reducing the occurrence of toxic metabolites, especially the oxalic acid, were reduced.

Fomepizole is also a potent inhibitor of alcohol dehydrogenase. Being similar to ethanol, it acts in blocking the formation of toxic metabolites. Fomepizole has been shown to be highly effective as an antidote for ethylene glycol poisoning. The American Academy of Clinical Toxicology recommends fomepizole in patients with ethylene poisoning in early stages to inhibit the alcohol dehydrogenase [**13**]. In Romania, this treatment is unavailable for the patients, because this medicine is not marketed.

Often, both the antidotal treatment and hemodialysis are used together in the treatment of poisoning. Because hemodialysis will also remove the antidotes from the blood, doses of antidotes need to be increased in order to compensate.

In addition to hemodialysis and the administration of antidotal treatment, metabolic acidosis can be corrected by the administration of sodium bicarbonate. In our cases, all the patients who had a Ph under the normal range (reference range = 7.35-7.42), received sodium bicarbonate, 1-2 mEq/kgb in addition to the specific treatment.

## Conclusions

Acute ethylene glycol poisoning has several clinical evolutions through the accumulation of toxic metabolites such as glycolic acid and oxalic acid. The early diagnostic and exclusion of other diseases and other poisoning led to a specific treatment of intoxication.

The time from the ingestion of ethylene glycol and early establishment of therapy is very important for a favorable evolution and can prevent substantial mortality. Patients presenting late with signs and symptoms of coma, hyperkalemia, seizures, or severe acidosis have a poor prognosis [**11**]. Patients with ethylene glycol intoxication are critical patients from the beginning and this life-threatening situation must be early recognized with the establishment of an emergency treatment.
